# RELATIONSHIP BETWEEN FAMILY INVOLVEMENT IN CARE PLANNING AND ESTABLISHING AN ADVANCE DIRECTIVE

**DOI:** 10.1093/geroni/igz038.2839

**Published:** 2019-11-08

**Authors:** Ellen McCreedy, lacey Loomer, Jennifer Palmer, Angelo Volandes, Susan Mitchell, Vincent Mor

**Affiliations:** 1 Brown University, School of Public Health, Providence, Rhode Island, United States; 2 Hebrew SeniorLife, Institute for Aging Research, Roslindale, Massachusetts, United States; 3 Harvard Medical School, Boston, Massachusetts, United States; 4 Hebrew SeniorLife, Institute for Aging Research, Boston, Massachusetts, United States

## Abstract

The purpose of this research is to understand the effect of family participation in care planning assessments on the use of advance directives for newly admitted nursing home (NH) residents. This is a retrospective cohort study using data from 115 nursing homes involved in a pragmatic randomized clinical trial testing a video intervention for advance care planning (versus usual care). Data sources included the electronic health record and the Minimum Data Set (MDS). Competing risks regression analyses estimated the cumulative incidence of establishing an advance directive, after accounting for death, discharge from NH, and time censoring (12-month observation window). 18,978 residents admitted to eligible NHs without an advance directive (full code) between April 2, 2016 and August 31, 2017 were followed for one year. 11,905 (63%) died or were discharged without an advance directive; 4,155 (22%) remained in the NH without an advance directive; and 2,918 (15%) established an advance directive. Median time to establishing an advance directive was 75 days (SD: 72, 79). After adjusting for competing risks and resident factors known to be associated with advance directive use, each care planning assessment involving a family member was associated with a 6% increase in the cumulative incidence advance directive use. Involving family members of NH residents in routine care planning assessments may reduce the amount of futile care received by NH residents. As we test interventions to improve advance care planning for NH residents, focus should be placed upstream on the first 90 days after admission.

